# The Involvement of Neonatal Intensive Care Unit and Other Perinatal Factors in Postpartum PTSD After Cesarean Section

**DOI:** 10.34763/jmotherandchild.20232701.d-23-00056

**Published:** 2023-11-03

**Authors:** Eirini Orovou, Panagiotis Eskitzis, Irina Mrvoljak-Theodoropoulou, Maria Tzitiridou-Chatzopoulou, Christiana Arampatzi, Nikolaos Rigas, Ermioni Palaska, Maria Dagla, Maria Iliadou, Evangelia Antoniou

**Affiliations:** Department of Midwifery, University of West Attica, Egaleo, Greece; Department of Midwifery, University of Western Macedonia, Keptse, Ptolemaida, Greece; Department of Psychology, National and Kapodistrian University of Athens, Athens, Greece

**Keywords:** Neonatal intensive care unit, NICU, postpartum PTSD, birth trauma, cesarean section, hospitalised neonate, traumatic birth experience

## Abstract

**Background:**

The experience of a neonate hospitalised in the Neonatal Intensive Care Unit (NICU) is an understandably traumatic experience for the parents, especially, for the mothers of neonates. This mental distress resulting from preterm birth and/or NICU hospitalisation can be understood as post-traumatic symptomatology, according to the Diagnostic and Statistical Manual-5 version. The aim of this study is to investigate the impact of the admission of a neonate to the NICU (from any reason) on the development of postpartum post-traumatic stress disorder (PTSD) in a sample of women after cesarean sections.

**Material and methods:**

A total of 469 women who gave birth with cesarean section from July 2019 to June 2020 participated in this study, from the original sample of 490 women who consented to participate. Data were obtained from the researcher's socio-demographic questionnaire, the past traumatic Life Events Checklist, the perinatal stressor Criterion A, and the Post-Traumatic Stress Checklist from the Diagnostic and Statistical Manual-5 version.

**Results:**

A percentage of 46.64% of sample experienced postpartum PTSD. Factors associated with PTSD were placenta previa type4, abruption, bleeding (β = .07, p = .049), premature contractions (β = .08, p = .039), heavy medical history or previous gynecological history and preeclampsia (β = .08, p = .034), abnormal heart rate, premature rupture of membrane, premature contractions, infections (β = .14, p = .004), life of child in danger (β = .12, p = .025), complications involving child (β = .15, p = .002), complications involving both (child and mother) (β = .12, p = .011), traumatic cesarean section (β = .041, p < .001) and prematurity (β = .12, p = .022).

**Conclusions:**

Additional measures must be taken for mothers of children who have been admitted to the NICU with psychological support interventions and reassessment of their mental state.

## 1 Introduction

The experience of a neonate hospitalised in the Neonatal Intensive Care Unit (NICU) is an understandably traumatic experience for the parents, while the feelings of mental distress may persist even after the neonate leaves the NICU [1]. Parents face separation from their child in an unfamiliar and stressful environment with access difficulties and sparing of communication and information from the staff [2]. Such an experience can affect parental mental health, with an impact on the transition to parenthood.

Mental distress resulting from preterm birth and/or NICU hospitalisation can be understood as post-traumatic symptomatology [3]. Post-traumatic stress disorder (PTSD) symptoms include reexperiencing the event, increased reactivity and avoidance and negative mood, while at least one month must have passed since the traumatic event [4] according to the Diagnostic and Statistical Manual DSM-5 [5]. It is already known that one in three women experience their delivery as a birth trauma [6], while one in four of them can develop postpartum PTSD (P-PTSD) [7]. Phillips [8] underlines the importance of the ‘magic hour’, that is, the first minutes immediately after birth in the mother–infant bond. Maintaining this contact after birth facilitates this bond and prevents the development of P-PTSD. Nevertheless, P-PTSD can affect the mother–infant bond [9, 10] and increase the infants' risks of developing behavioural or emotional problems later [11].

Several studies have demonstrated that many stressful events during the perinatal period, such as pathology of gestation [12], emergency cesarean delivery [13, 14], preterm birth [15] and postpartum complications [14] contribute to the development of P-PTSD [16]. Especially, an emergency cesarean section (CS) has been linked to increased rates of maternal depression and PTSD in the postpartum period [17, 18, 19], due to the unexpected event and the lack of the mother's mental preparation [20].

To meet the diagnosis of PTSD, exposure to a potentially stressful factor (Criterion A) must apply. More specifically, there must be exposure to actual or threatened death, serious injury or sexual violence in any of the following ways: a) direct exposure, b) as a witness to the event, c) learning that the traumatic event happened to a significant other and d) daily exposure through work [21]. Based on the above, admission to the NICU is considered a traumatic event for mothers, who, in addition to a potentially traumatic birth experience, such as CS delivery, face the threat of death or physical unseemliness of the neonate.

On the other hand, the prevalence of PTSD in mothers of infants admitted to the NICU ranges between 24% and 44%, according to previous studies [22, 23]. So far, however, NICU admission has not been studied as an additional trauma variable in postpartum women. Furthermore, it is not known to what extent the NICU may contribute to the high rates of PTSD in women after CS. Given that women who undergo CS (emergency or elective) are more likely to develop post-traumatic symptoms [13, 14, 17, 24,], the admission of the neonate to the NICU of CS-exposed women makes them a particularly vulnerable group of postpartum mothers. So far, there are several studies that show a correlation between CS and PTSD [17, 24]. It has also found a positive relationship of the admission of the newborn to the NICU [25, 26]. However, there is no study that identifies the effect of the NICU on the special group of women after CS.

Therefore, the purpose of this study is to investigate the involvement of the admission of a neonate to the NICU, and other perinatal factors on the development of P-PTSD in a sample of women after CS.

## 2 Material and Methods

The specific study was carried out between July 2019 and June 2020 at the obstetrics clinic of the University Hospital of Thessaly in Greece with a prospective design. Our initial sample consisted of postpartum women after CS, aged 18 or older, who gave written informed consent to participate in the study. Finally, 469 postpartum women responded to the follow-up and constituted the sample of our study. Ethics Commission, Approval: 18838/08-05-2019, University of Thessaly.

### 2.1 Research participation criteria

The research participants who gave birth with CS had a complete medical record at the specific hospital, which means that they were monitored during the entire pregnancy or during most of it at the hospital, had a sufficient understanding of the Greek language, satisfactory mental level and were not taking psychotropic drugs or other substances during the perinatal period. Those postpartum women who did not meet the above criteria were excluded from the study.

### 2.2 Measures and data

Data were collected in two phases. During the first phase, questionnaires were given by the researcher to postpartum women on the second day after CS delivery during their hospitalisation (socio-demographic, Life Events Checklist Criterion A), after their informed consent, while data on each woman's medical history were obtained. The second phase took place during the sixth week postpartum. Through a telephone interview, the postpartum women answered questions about the existence of post-traumatic symptoms according to the PTSD scale (PCL-5). In order to be diagnosed with PTSD, it is necessary that a month has passed since the traumatic event, so this specific period (6^th^ week) was considered appropriate to meet the diagnostic criteria [21]. All measures were received from the National Center of PTSD [4] and were translated and weighted into the Greek language by two research members (E.O. and E.A.). For the needs of this research, the van de Vijver & Leung [27] method was used, in which two bilingual independent obstetric care professionals translated the questionnaires from their original version (English language) into Greek. Then, the two versions were compared, and minor modifications were made to arrive at the final form of the psychometric instruments used in the research. This final form was retranslated by a bilingual healthcare professional (with American and Greek citizenship), from the Greek language to their original English form, and then the final form of the translated questionnaires were compared with the originals, without the research team intervention. Therefore, the final forms of the translated scales resulting from the above procedure constituted the measurement scales used in this research. Subsequently, the set of questionnaires was pilot administered to a sample of 23 postpartum women before starting the main survey. The aim of the pilot administration of the questionnaires was to check the degree of understanding of the questions by all women regardless of age, socio-economic status and educational level. Regarding the psychometric properties of the tools, the PCL-5 showed very good psychometric properties during its evaluation. More specifically, the Cronbach's α of this scale in our sample is .97 [28]. The Cronbach's α of Criterion A regarding our sample is .92, and correspondingly, the Cronbach's α of the LEC-5 scale is .69.

#### 2.2.1 A researcher socio-demographic questionnaire

This questionnaire included sections on the woman's social characteristics, demographic data and medical history that included obstetric, gynecological psychiatric and family information concerning the present situation, the neonate and finally, specialised questions about the experience of birth with CS.

#### 2.2.2 Past traumatic life events by Life Events Checklist [29]

The Life Events Checklist (LEC-5) investigates an individual's potential exposure to 16 traumatic events that may lead to PTSD or distress. The LEC-5 cannot be scored; it simply identifies traumatic events in a person's life.

A unique feature of the LEC-5 is that respondents can simultaneously indicate different levels of exposure to a traumatic event (happened to me, saw it, learned about it, part of my job, not sure, not true).

#### 2.2.3 Stressor perinatal Criterion A

Perinatal Criterion A is based on the PTSD Criterion A [30], according to the DSM 5. Criterion A is the first criterion, the existence of which is considered necessary for the other criteria to appear: Criterion B: Re-experiencing, Criterion C: avoidance, Criterion D: negative alterations in cognitions and mood and Criterion E: hyperarousal and reactivity [4]. For the purposes of this study, Criterion A examined population exposure of women following emergency cesarean section (EMCS) and elective cesarean section (ELCS). Therefore, Criterion A was adapted with appropriate specialised questions that diagnosed exposure to a maternal or foetal/neonatal life-threatening traumatic event. However, the perinatal stressor Criterion A was divided into: a) Criterion A1, which detected maternal or child-threatening situations during or shortly before CS; and b) Criterion A2, which detected complications in mother–child life after CS.

#### 2.2.4 Post-Traumatic Stress Checklist (PCL-5)[31]

It is a scale that assesses 20 post-traumatic symptoms according to the DSM-5 and provides a provisional PTSD diagnosis. Postpartum women were asked to answer 20 self-report items that assessed 20 PTSD symptoms of the criteria: B (reexperiencing), C (avoidance), D (negative thoughts and feelings), and E (arousal and reactivity). If, in addition to Criterion A, Criteria B, C, D, and E are met, the provisional diagnosis of P-PTSD is considered certain [32].

### 2.3 Statistical Analysis

Quantitative variables are expressed as mean values (standard deviation [SD]) or as median values. For comparisons of proportions, chi-squared and Fisher's exact tests were used. Logistic regression analyses were performed to identify admission to NICU associated with the presence of PTSD or PTSD profile in postpartum women. Unadjusted and adjusted odds ratios with 95% confidence intervals were computed from the results of the logistic regression analyses. Statistical significance was set at 0.05, and analyses were conducted using SPSS statistical software (SPSS Statistics version 22.0, IBM, Armonk, NY, USA).

## 3 Results

A total of 469 women were analysed in this study. Their mean age was 32.58 ± 6.15 (*SD*) years, with the majority of them being from the city (*N* = 373, 79.5%); 98.5% (*N* = 462) were married, engaged or in a relationship. Overall, 43.4% (*N* = 204) of the sample had completed undergraduate and/or postgraduate studies, with a similar percentage who had finished high school (*N* = 196, 41.8%); 10.7% (*N* = 69) participants had finished primary school or junior high. None of these demographic variables or the occupation of the participants relate to PTSD. However, it seems that the financial status contributes to PTSD, but without an adequate number in the last category (high), preventing us from assumptions about differences between categories (Table 1). For almost all independent variables presented in Table 1, and PTSD as dependent, one-way analyses of variance (ANOVA) were applied.

**Table 1.** Demographic, perinatal and mental health variables in relationship to PTSD

**Demographic Variables**	**f/M**	**rf/SD**	***p***
**Age**	32.58	6.15	.445^a^
Residence			.921^b^
City	373	79.5	
Village	96	20.5	
Total	469	100.0	
Nationality			.447^b^
Greek	423	90.2	
Other	46	9.8	
Total	469	100.0	
Family Status			.239^b^
Single /Divorced	7	1.5	
In Relationship/Engaged	47	10.0	
Married	415	88.5	
Total	469	100.0	
Educational Level			.193^b^
Primary School	39	8.3	
Junior High	30	6.4	
High School	196	41.8	
University	170	36.2	
MSc/PhD	34	7.2	
Total	469	100.0	
Occupation			.181^b^
Public/Private Sector	136	29.0	
Freelance	67	14.3	
Healthcare Professional	38	8.1	
Educators	43	9.2	
Household	123	26.2	
Unemployed	62	13.2	
Total	469	100.0	
Financial Status			.014^b^
Low	136	29.0	
Middle	320	68.2	
High	13	2.8	
Total	469	100.0	
*Perinatal Health Variables*			
Parity			.013^b^
0	202	43.1	
1 birth	177	37.7	
≥ 2 births	90	19.2	
Total	469	100.0	
Type of Previous Labour			*p* < .001^b^
No Previous Labour	204	43.5	
Vaginal	41	8.7	
C-section	224	47.8	
Total/Missing	469	100.0	
Kind of Conception			.490^b^
Normal	429	91.5	
In Vitro Fertilization (IVF)	40	8.5	
Total	469	100.0	
Gynecologic History			.012^b^
No	421	89.8	
Yes	48	10.2	
Total	469	100.0	
Gestational Week	37.76	2.10	*p* < .001^b^
< 37	70	14.9	
≥ 37	399	85.1	
Total	469	100.0	
Type of C-section			*p* < .001^b^
Emergency	181	38.6	
Elective	288	61.4	
Total	469	100.0	
Cause of C-section			*p* < .001^b^
Previous C-section/Premature Rupture of Membranes/Premature Contractions in a Previous C-section/Placenta Previa	203	43.3	
Abnormal Foetal Position	66	14.1	
Twins/IVF gestation	30	6.4	
Mother's Desire	30	6.4	
Heavy Medical History/Myopia/Previous Gynecological History/Preeclampsia	35	7.5	
Failure of Labour to Progress	43	9.2	
Abnormal Heart Rate/Pathological NST/Doppler/Premature Rupture of Membranes/Premature Contractions/Infection	62	13.2	
Total	469	100.0	
C-section Complications			*p* < .001^b^
No	434	92.5	
Yes	35	7.5	
Total	469	100.0	
Birth Expectations/Satisfaction			*p* < .001^b^
No	245	52.2	
Yes	224	47.8	
Total	469	100.0	
Admission to Neonatal Intensive Care Unit (NICU)			*p* < .001^b^
No Admission to the NICU	367	78.3	
Perinatal Stress/Breathing Difficulty/Infection/IUGR/Other Disorders	53	11.3	
Prematurity	49	10.4	
Total	469	100.0	
Breastfeeding			*p* < .001^b^
No	153	32.6	
Yes	316	67.4	
Total	469	100.0	
*Mental Health Variables*			
Psychiatric History			*p* < .001^b^
No	411	87.6	
Yes	58	12.4	
Total	469	100.0	
Partner Support			*p* < .001^b^
No	68	14.5	
Yes	401	85.5	
Total	469	100.0	
Traumatic C-section			*p* < .001^b^
No	230	49.0	
Yes	239	51.0	
Total	469	100.0	
Criterion A1 – Was your life or your child's life in danger?			*p* < .001^b^
No	368	78.5	
Yes, of My Child	63	13.4	
Yes, Mine	17	3.6	
Yes, of Both of Us	21	4.5	
Total	469	100.0	
Criterion A2 – Any complications involving you or your child?			*p* < .001^b^
No	400	85.3	
Yes, My Child	43	9.2	
Yes, Me	16	3.4	
Yes, Both of Us	10	2.1	
Total	469	100.0	
Diagnosis			*p* < .001^b^
No Diagnosis	378	80.6	
Partial PTSD	36	7.7	
PTSD	55	11.7	
Total	469	100.0	
No Admission to the NICU			
No Diagnosis	318	86.6	
Partial PTSD	33	9.0	
PTSD	16	4.4	
Total	367	100.0	
Perinatal Stress/Breathing Difficulty/Infection/IUGR			
No Diagnosis	31	58.5	
Partial PTSD	2	3.8	
PTSD	20	37.7	
Total	53	100.0	
Prematurity			
No Diagnosis	29	59.2	
Partial PTSD	1	2.0	
PTSD	19	38.8	
Total	49	100.0	

*Note:*

a – Pearson*r*,

b – ANOVA,

f = -value in analysis of variance,

M = mean value.

Taking into consideration perinatal and mental health variables from Table 1, except for a kind of conception variable, a statistically significant relationship with PTSD can be noted. Therefore, multiple analyses revealed that parity, type of previous labour, gynecologic history, gestational week, type of C-section, cause of C-section, C-section complications, birth expectations, admission to the NICU, breastfeeding, psychiatric history, partner support and traumatic C-section, as well as Criterion A1 and A2 variables, were independently associated with the presence of PTSD.

The next analysis conducted in the present study, with the independent variable admission to the NICU and the dependent PTSD, is univariate ANOVA, while for the independent variables being the four factors of PCL-5 (intrusion/re-experiencing, avoidance, negative alterations in cognitions and mood & alterations in arousal and reactivity) multivariate ANOVA was applied. Prior to this, the correlations analysis among PCL-5 factors showed high, positive correlations, ranging from .717 to .919. The results of the ANOVAs are presented in Table 2. The cutoff for the provisional PTSD diagnosis was 33.

**Table 2.** The results of the analyses of variance

	**Admission to the NICU**						
	**No Admission to the NICU**	**Perinatal Stress/Breathing Difficulty/Infection/IUGR**	**Prematurity**				

	**M**	**M**	**M**	**F**	**df**	***p***	**η^2^**
PTSD	6.18a	20.64b	21.24b	46.64	2	*p* < .001	.388
Intrusion/Re-experiencing	1.68a	5.75b	7.18b	56.31	2	*p* < .001	.195
Avoidance	.72a	2.26b	2.20b	31.58	2	*p* < .001	.119
Negative Alterations in Cognitions and Mood	2.34a	7.04b	6.71b	32.28	2	*p* < .001	.122
Alterations in Arousal and Reactivity	1.44a	5.58b	5.14b	41.31	2	*p* < .001	.151

*Note:* Means that share a common index (b) do not differ significantly from each other according to the Scheffé post-hoc criterion.

At the univariate level, the *F* criterion showed a statistically significant relationship between admission to the NICU and all five independent variables. According to the Scheffé post-hoc criterion, it is observed that mothers of the present sample, whose child was admitted to the NICU, either because of prematurity or any other reason reported in Table 2, are more likely to develop PTSD (*p* < .001), as well as to have higher scores on the intrusion/re-experiencing (*p* < .001), avoidance (*p* < .001), negative alterations in cognitions and mood (*p* < .001), and in alterations in arousal and reactivity subscale (*p* < .001).

Finally, a multiple linear regression analysis was performed in order to investigate the relation between the reasons for the admission to the NICU, in correlation to pathology gestation, cause of C-section and Criterions A1 and A2, and PTSD as a dependent variable. Therefore, binary basis variables, also called dummy variables, are created to represent the independent variables with two or more distinct categories included in the present analysis; categorical independent variables cannot be directly entered into a multiple regression analysis. Results are presented in Table 3, as well as categories of the intercorrelated variables. Twenty-one independent variables were included in the analysis, as if they were all potential predictors of PTSD.

**Table 3.** Results of multiple regression analyses with presence of PTSD as a dependent variable.

	***b***	***S.E.***	**β**	***t***	***p***
(Constant)	.19	.09		2.15	.032
*Pathology of Gestati*on					
Thrombophilia/Hyperemesis	.27	.42	.02	.63	*ns*
Placenta Previa Type4/Abruption/Bleeding	.55	.28	.07	1.98	.049
Diabetes	.21	.17	.05	1.26	*ns*
Cervical Insufficiency	.80	.41	.07	1.94	*ns*
Infection	.60	.49	.05	1.24	*ns*
Premature Contractions	.84	.40	.08	2.07	.039
*Cause of C-section*					
Abnormal Foetal Position	.11	.16	.03	.65	*ns*
Twins/IVF Gestation	.09	.22	.01	.39	*ns*
Mother's Desire	.19	.22	.03	.86	*ns*
Heavy Medical History/Previous Gynecological History/Preeclampsia	.49	.23	.09	2.13	.034
Failure of Labour to Progress	−.05	.19	−.01	−.26	*ns*
Abnormal Heart Rate/Pathological NST/Premature Rupture of Membrane/Premature Contractions/Infection	.61	.21	.14	2.87	.004
*Criterion A1 & A2*					
Life of Child in Danger	.50	.22	.12	2.24	.025
Life of Mother in Danger	.32	.34	.04	.95	*ns*
Both Lives in Danger	.69	.37	.10	1.89	*ns*
Complications Involving Child	.77	.25	.15	3.11	.002
Complications Involving Mother	.52	.35	.07	1.49	*ns*
Complications Involving Both (Child and Mother)	1.19	.46	.12	2.56	.011
Traumatic C-section	1.19	.12	.41	9.55	*p* < .001
*Reasons for the NIC*U					
NICU – Perinatal Stress/Breathing Difficulty/Infection/IUGR	.02	.22	.01	.08	*ns*
NICU – Prematurity	.57	.25	.12	2.29	.022

*Note: R* = .466, *R*^2^ = .440, *F* = 17.69, *df* = 22, *p* < .001

Through the linear regression, with PTSD being the dependent variable relating to the 21 perinatal health independent variables, the model introduced here has only nine predictors that were strongly related to PTSD: placenta previa type4/abruption/bleeding (β = .07, *p* = .049), premature contractions (β = .08, *p* = .039), heavy medical history/previous gynecological history/preeclampsia (β = .08, *p* = .034), abnormal heart rate/pathological NST/premature rupture of membrane/premature contractions/infection (β = .14, *p* = .004), life of child in danger (β = .12, *p* = .025), complications involving child (β = .15, *p* = .002), complications involving both (child and mother) (β = .12, *p* = .011), traumatic C-section (β = .041, *p* < .001) and prematurity (β = .12, *p* = .022). The regression equation reached significance, signifying that 46.64% of the variance of PTSD could be explained by this regression model. Consequently, the participants who experienced any of the situations appearing as a predictor in the present model were more likely to develop PTSD.

## 4 Discussion

According to our results, 46.64% of postpartum women after CS experienced P-PTSD. The high rate of PTSD is consistent with an earlier study in which PTSD was present in 60% of mothers whose children were admitted to the NICU [33]. Also, the results of another study [23] showed that the acute PTSD symptoms of postpartum mothers were related to having a neonate in the NICU.

Several studies identified cesarean delivery as a major factor of P-PTSD [17, 24, 34], especially emergency CS [13, 24, 35]. In addition, it was found that P-PTSD was associated with placenta previa (type 4), abruption, bleeding, premature contractions, infections and preeclampsia. It is already known that bleeding significantly increases the risk of PTSD [36, 37], since bleeding is a traumatic event for the mother, as an obstetric emergency with a possible risk of losing her life or the life of the foetus. Premature contractions or premature rupture of membranes and preeclampsia increase the risk of preterm or complicated birth [38, 39] and increase the risk of development P-PTSD [40]. Also, conditions that may lead to preterm birth, such as infections, are also responsible, according to our findings, for the development of P-PTSD [41].

Our results show that previous heavy medical history or gynecological history, associated with P-PTSD. One explanation for this phenomenon is that women's gynecological history may affect the course of pregnancy and, therefore, create complications related to the health of the neonate. However, abnormal foetal heart rate during labour increases the chance of an emergency CS [42].

One of the important findings of this study related to P-PTSD was the fulfillment of the perinatal stressor Criterion A. More specifically, all life-threatening situations of mother, child, or both, before delivery, during CS, or after are development factors of P-PTSD. However, all of the above factors, including the inclusion of a neonate in NICU, for any reason are considered risk factors for P-PTSD [43].

P-PTSD of mothers who have children in the NICU is multifactorial. Two important factors that may play a decisive role in the emergence of post-traumatic symptomatology are the severity of the neonatal illness and the parents' perception of the severity of the illness [44]. Also, within the NICU environment, the maternal role is diminished, as there are strong restrictions governing the nursing and operating regulations of the unit. Therefore, mothers may perceive the infant's hospitalisation as a threat of death or disability, and furthermore, images within the incubator may further reinforce these fears [45]. As a result, prolonged mental distress in mothers often disrupts the mother–infant relationship and also appears to affect children's psychosocial development in the future [46, 47]. In addition, the removal of the neonate from the mother results in lack of breastfeeding, which increases the intensity of the traumatic experience and the emergence of post-traumatic symptoms [48].

## 5 Conclusions

According to our findings, the admission of a neonate to the NICU is an important factor in the development of P-PTSD in women after CS. Also, the perinatal factors that lead a neonate to NICU were identified as dangerous for the occurrence of P-PTSD, since they indicate the intense stress on the part of the mother for the risk of losing the life of the neonate. Therefore, it is very important that special groups of women who have undergone a CS, especially an emergency one, are treated as individuals who may develop PTSD in the postpartum period. Additional measures must be taken for mothers of children who have been admitted to the NICU with psychological support interventions and reassessment of their mental state. Special care is required to strengthen breastfeeding, in order to reduce the intensity of the birth trauma and develop the mother–child bond.
